# Endosperm-specific expression of human acid beta-glucosidase in a waxy rice

**DOI:** 10.1186/1939-8433-5-34

**Published:** 2012-12-06

**Authors:** Tamara Patti, Bruno Bembi, Piero Cristin, Flavia Mazzarol, Erika Secco, Carla Pappalardo, Rita Musetti, Maurizio Martinuzzi, Serena Versolatto, Roberta Cariati, Andrea Dardis, Stefano Marchetti

**Affiliations:** Transactiva Srl, Via J. Linussio 51, 33100 Udine, Italy; Regional Coordination Centre for Rare Diseases, University Hospital S. Maria Misericordia, P.zale S. Maria della Misericordia 15, 33100 Udine, Italy; Department of Agriculture and Environmental Sciences, University of Udine, Via delle Scienze 206, 33100 Udine, Italy

**Keywords:** Human acid beta-glucosidase, Gaucher disease, Transgenic rice, Pharmaceutical proteins, Endosperm-specific expression, Molecular farming

## Abstract

**Background:**

The deficiency of human acid beta-glucosidase (hGCase) causes Gaucher disease, a rare genetically-inherited disorder currently treated by enzyme replacement therapy using recombinant CHO-derived GCase. In an attempt to provide an alternative and more efficient production system, a chimeric cDNA coding for hGCase operatively linked to the signal peptide of rice glutelin 4 (GluB4) was put under the control of the GluB4 endosperm-specific promoter and inserted into the genome of a waxy rice.

**Results:**

Molecular, immunological and biochemical analyses showed that recombinant hGCase, targeted to the protein storage vacuoles of rice endosperm cells, is equivalent to the native protein and has a glycosylation pattern compatible with direct therapeutic use. Compared to a previous study carried out on transgenic tobacco seeds, enzyme contents per unit of biomass were drastically increased; in addition, differently from what observed in tobacco, rice seed viability was unaffected by hGCase even at the highest production level. Transgenic seed polishing combined with a pretreatment of seed flour greatly facilitated hGCase extraction and purification with an industrially-scalable procedure.

**Conclusions:**

This study opens up the possibility to efficiently produce in the rice seed pharmaceutical compounds which are available in limited amounts or completely excluded from clinical practice due to the inadequacy of their production systems.

**Electronic supplementary material:**

The online version of this article (doi:10.1186/1939-8433-5-34) contains supplementary material, which is available to authorized users.

## Background

Human acid beta-glucosidase (hGCase; EC 3.2.1.45) catalyzes the hydrolytic cleavage of glucose from glucosylceramide in lysosomes (Brady et al.^1965^). It is a homomeric glycoprotein of 497 amino acids with a molecular mass ranging from 60 to 68 kDa, containing five putative N-glycosylation sites, four of which are normally occupied (Berg-Fussman et al.^1993^; Brumshtein et al.^2006^). The quantitative/qualitative deficiency of hGCase leads to an accumulation of glucosylceramides in lysosomes and causes the Gaucher disease (GD), one of the most frequent human sphingolipidosis. Fatty material can collect in the spleen, liver, kidneys, lungs, brain and bone marrow. Main clinical symptoms may include hepatosplenomegaly, anaemia, thrombocytopenia and skeletal deterioration (2001). GD has a pan-ethnic diffusion but its frequency is particularly high (1:640 to 1:10,000) in the Ashkenazi Jewish population (Beutler and Grabowsky^2001^;^1997^), whereas in the non-Jewish population data are varying from 1:57,000 to 1:200,000 (Cox and Schofield^1997^; Meikle et al.^1999^).

GD is currently treated by enzyme replacement therapy (ERT) using mainly a recombinant processed form of human acid beta-glucosidase (commercial imiglucerase) obtained from cultured CHO cells. Regular infusions of the missing enzyme were found effective in reducing the visceral and bone marrow storage of glucosylceramides, decreasing spleen and liver volumes, normalizing/stabilizing hematochemical parameters (Hb, platelets, hepatic enzymes, chitotriosidase) and improving the quality of life of GD patients (Grabowski et al.^1995^; Damiano et al.^1998^). However, the current production system based on CHO is economically very expensive and limits the drug amounts present on the market, seriously hindering the maintenance and the diffusion of therapeutic regimes.

Moreover, an acute shortage of commercial imiglucerase has recently occurred as a result of viral contamination of the production facility, forcing all countries to reassess their stocks and to establish new priorities of drug supply to patients (Hollak et al.^2009^).

Although an additional two enzymatic forms were approved for early access clinical programs, i.e. velaglucerase alpha (from cultured human fibroblasts; Aerts et al.^2010^) and taliglucerase alpha (from cultured carrot cells; Shaaltiel et al.^2007^, Aviezer et al.^2009^), the drug shortage has underlined the necessity to find a valid alternative production system to guarantee sufficient drug supply to patients not only for Gaucher disease, but also for other disorders and diseases curable with lifelong therapies.

Molecular farming, i.e. the production of valuable recombinant proteins in plants (Schillberg et al.^2003^), can represent a good solution to this need, because plants have several advantages over traditional platforms for recombinant pharmaceutical protein production. Plants are inexpensive, they are highly scalable, they offer high product quality and they do not support human pathogens. Crops producing pharmaceutical molecules can be established with minimal upfront investment in infrastructures, unlike the major fermentation-based platforms (Fischer et al.^2012^).

Concerning Gaucher disease, in a previous study, a procedure for production and immuno-purification of human acid beta-glucosidase in tobacco seeds was developed (Reggi et al.^2005^). The plant-derived enzyme was found stable, active and taken up by human target cells; nevertheless, it was also found that the best transgenic lines could not be maintained through sexual reproduction due to seed non viability; this fact combined with the tiny dimensions and the high lipid content of tobacco seed had a negative impact on industrial scalability.

The present paper describes a novel technological platform for the large-scale production of recombinant human GCase (rhGCase) in the endosperm of *Oryza sativa* L. In the new system, seed germination is not impaired by the recombinant enzyme, protein extraction and purification can be easily performed with resins available in bulk amounts and the product quality appears largely suitable for therapeutic purposes.

## Results

### Rice transformation and molecular analyses on primary transformants

**Figure 1:**
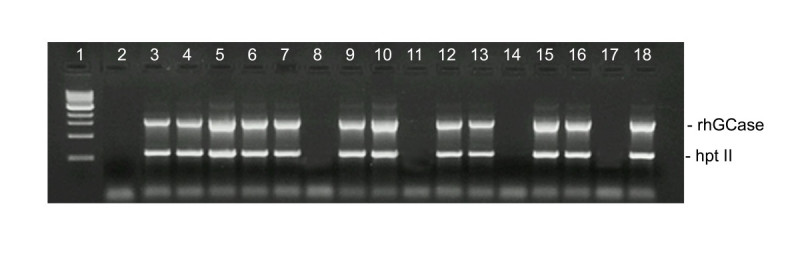
**Duplex-PCR products obtained using primers specific for** ***hGCase*** **and** ***hpt-II*** **(hygromycin resistance) genes.** Lane 1: 1 kb ladder (NEB); 2: negative control (untransformed CR W3); 3–18: tested plants.

Compared to other japonica varieties, the waxy rice CR W3 is less responsive *in vitro*, therefore preliminary experiments were done to select the best conditions for embryogenic callus formation and plant regeneration. *Agrobacterium*-mediated transformation was efficient only when scutellum-derived calli were less than 1 mm in diameter, non-friable, whitish and roundly shaped; moreover, such calli had to be used within 10 days after isolation and selection. Regenerated plants were analysed by PCR to verify the presence of both *hGCase* and selection marker genes (Figure 1). A total of 67 plants were found positive and used for further analyses. A sample of 16 randomly-chosen transformants was used to estimate the transgene copy number by real-time qPCR. In these experiments, the endogenous reference was the sucrose phosphate synthase (*SPS*) gene, which is present in a single copy in the haploid rice genome. Titration curves were obtained by regressing threshold cycles on known dilutions of *SPS* and *hGCase* genes; correlation coefficients between the two variables ranged from 0.985 to 0.993. PCR efficiencies were above 0.90. In the sample of primary transformants, transgene copy number was in most cases ≤ 3 and never higher than 4.

### Western blot analyses on seed protein extracts

**Figure 2:**
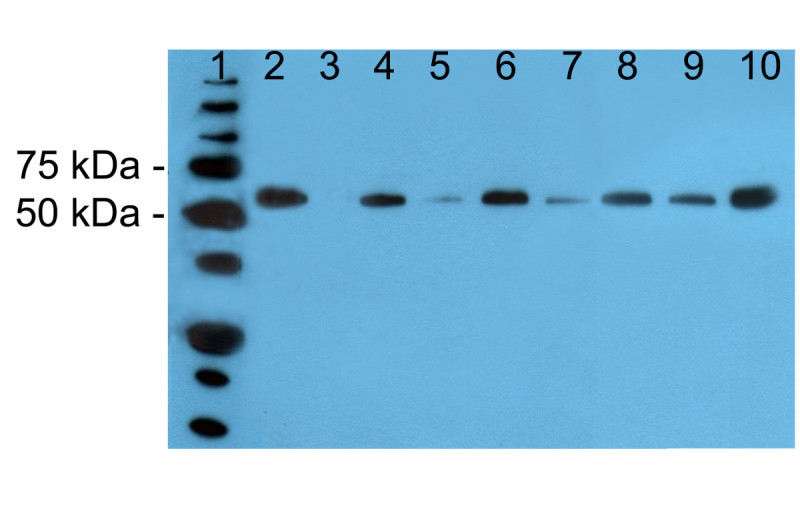
**Western blot analysis of crude seed protein extracts.** Lane 1: Precision Plus Protein standard (BioRad); 2: positive control (100 ng imiglucerase); 3: negative control (protein extract from untransformed CR W3 seed); 4–10: tested plants.

As formerly described, the human *GCase* gene was linked to an endosperm-specific rice glutelin (GluB4) promoter, enhanced by the substitution of its native leader region with the synthetic LLTCK 5’ UTR (De Amicis et al.^2007^). To confirm the endosperm expression of the GluB4 promoter, seed protein extracts were analyzed by Western blotting using the anti-GCase serum raised in rabbit after injection of the GCase-analogue imiglucerase. These analyses demonstrated that rhGCase is accumulated in the developing seed. No cross-reacting proteins were ever identified in seed protein extracts of untransformed control. In all transgenic samples, the antibody revealed a single protein band, having an apparent molecular weight of 60 kDa, that is very similar to commercial imiglucerase (Figure 2). Since the molecular mass of the unglycosylated protein is 55.6 kDa, this result indicated that rhGCase produced in the rice endosperm is glycosylated with low molecular weight N-glycans.

The Western blot signal intensity differed among lines; since in a previous study the band intensity was found correlated with rhGCase content (Reggi et al.^2005^), Western blotting was used to select the best primary transformants, until a consistent ELISA was developed. The protein extracts of 18 transformants repeatedly gave better results; this superiority was confirmed in Western blot analyses carried out using different sample dilutions. Best transformants were grown to produce a T_2_ generation for further quantitative analyses, whereas the seed of the remaining lines was bulked and used to develop the GCase purification procedure.

### Subcellular localization of recombinant GCase

**Figure 3:**
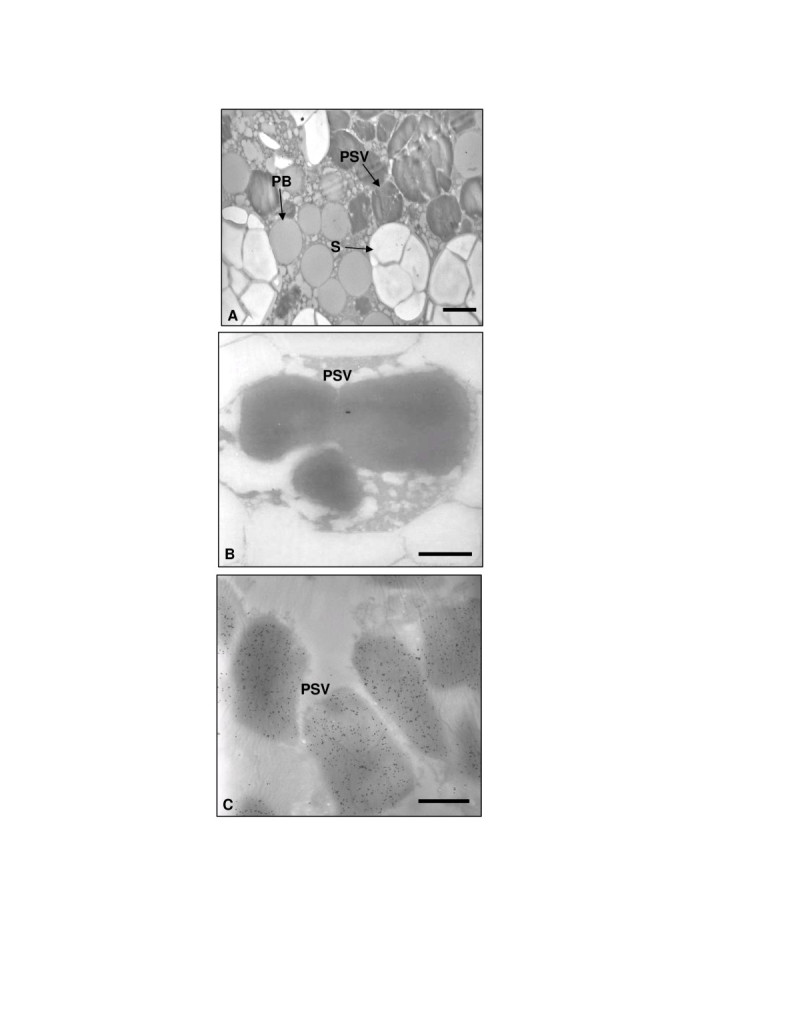
**Electron and immunoelectron microscopy on rice endosperm sections.** Micrograph of unlabelled osmium-treated sample (**A**), untransformed control (**B**) and transformed rhGCase seed (**C**). PSV: protein storage vacuole; PB: protein body; S: starch granule. Bars = 3 μm in (**A**), 1 μm in (**B**) and (**C**).

The glutelin 4 signal peptide was used with the expectation that rhGCase would be targeted to the protein storage vacuoles (PSVs) within the endosperm cells. To precisely determine the site of rhGCase accumulation and storage, transformed and untransformed seeds were repeatedly examined at the transmission electron microscope following immunogold labeling. Since this immunocytochemical technique does not preserve the ultrastructural morphology of the tissues, the main cellular ultrastructures of rice seed endosperm were preliminary identified in osmium-treated samples (Figure 3A). No signal was ever found in untransformed control seed; differently, in transgenic seed the presence of the recombinant enzyme was revealed exactly where it was planned, i.e. within protein storage vacuoles (PSVs) of the seed endosperm (Figure 3C); interestingly, no immunogold labeling was obtained in the protein bodies.

### Tissue-specific expression analyses

**Figure 4:**
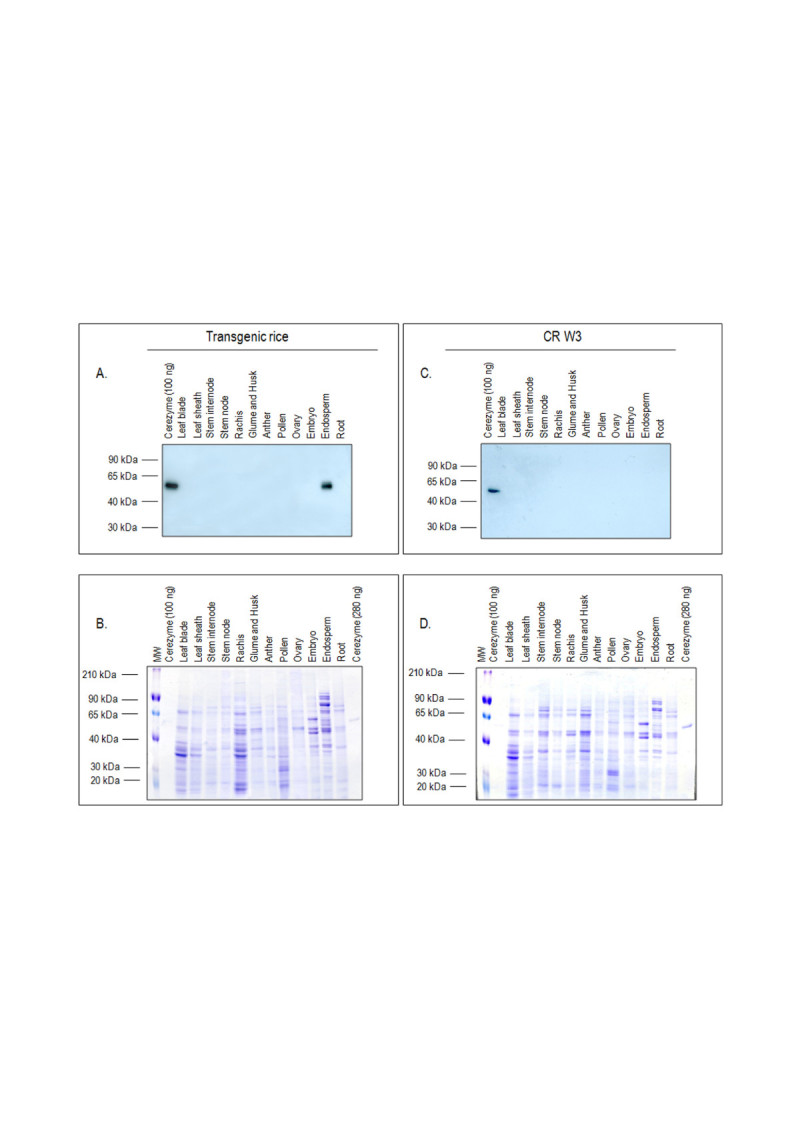
**Detection of rhGCase in protein extracts from different tissues of transgenic and wild type plants.** **A** and **C**: Western blot analyses performed using an anti-rhGCase polyclonal antibody; **B** and **D**: SDS-PAGE and Coomassie staining of the same protein samples of **A** and **C**, respectively.

Protein samples extracted from different tissues/organs showed the typical electrophoretic pattern; no difference was noted between the transgenic line and the CR W3 variety providing the genetic background (Figure 4B and4D).

Western blot analyses indicated the endosperm-specific expression of rhGCase (Figure 4A). No cross-reacting protein appeared in the negative control CR W3 (Figure 4C).

**Figure 5:**
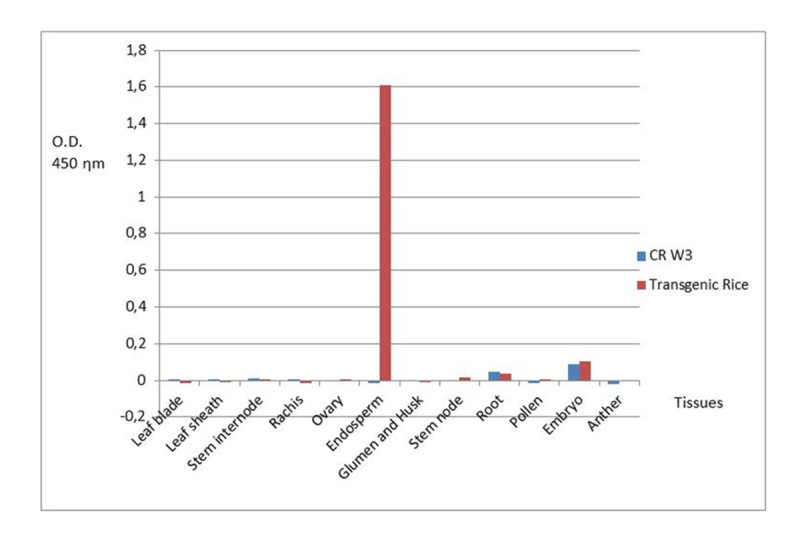
**Results of DAS-ELISA analyses carried out on protein samples obtained from different tissues of transgenic and wild type plants.** O.D.: optical density.

Results obtained with DAS-ELISA were consistent in showing the endosperm-specific expression of the *rhGCase* gene (Figure 5). Some background noise was evidenced for root and embryo protein extracts but the overall clearness of the data was not hampered in any respect.

### rhGCase extraction and purification

**Figure 6:**
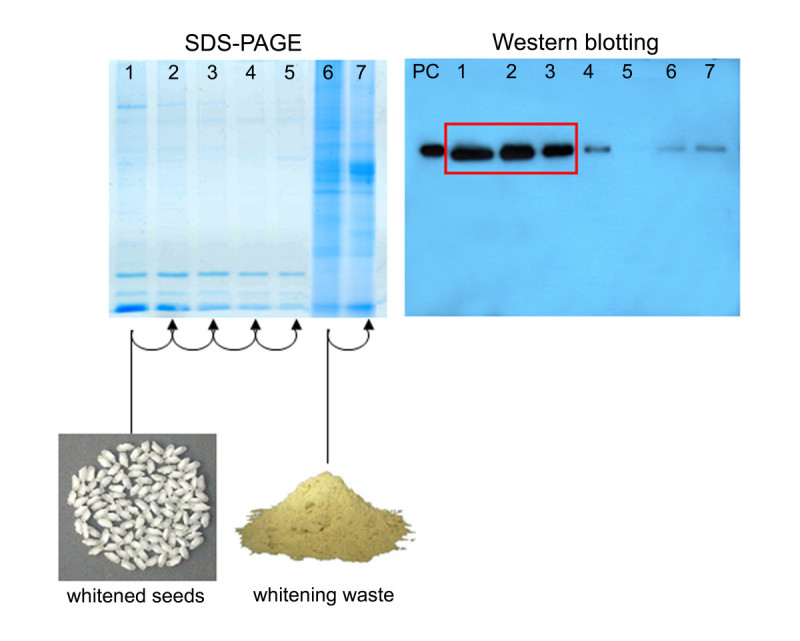
**SDS-PAGE and Western blot analyses of crude protein samples obtained during extraction trials.** Lanes 1–5: serial extractions from polished seed; lanes 6–7: serial extractions from polishing waste. PC (positive control): 100 ng imiglucerase.

On the basis of the endosperm-specific localization of rhGCase, a first trial took into consideration the possible advantage of seed polishing for the partial removal of protein contaminants. Transgenic white rice was milled and analysed in parallel with the polishing waste. From the combination of the results obtained in SDS-PAGE and Western blotting, it appeared that, differently from the large part of contaminants, only a minimal quantity of rhGCase is removed during processing (Figure 6). Subsequent experiments were designed to determine the conditions for rhGCase extraction from polished seed flour. Figure 6 shows the results obtained in 5 and 2 serial extractions carried out on the polished seed flour and the polishing waste, respectively. It is evident from the figure that most rhGCase is recovered from flour with the first three extractions and that in the fifth extract the enzyme is hardly detectable. Percent enzyme recovery as estimated by ELISA in the first, second and third extraction was 64.7, 22.9 and 7.2%, respectively.

DAS-ELISA was also used to determine the rhGCase content in polished rice flour derived from a bulk of unselected seeds harvested from the best primary transformants. The overall estimated yield was 143 mg of rhGCase per kg of flour.

**Figure 7:**
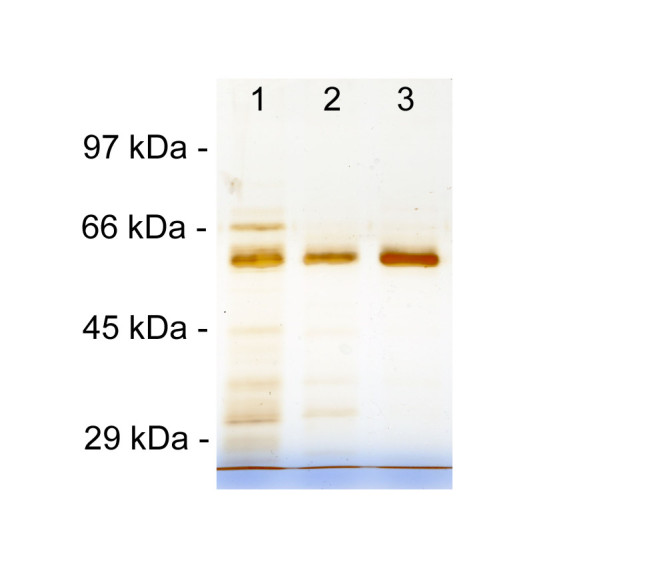
**SDS-PAGE analysis carried out to estimate contaminant removal obtained in each chromatographic step.** Lane 1: HIC (Hydrophobic Interaction Chromatography) eluate; 2: IEC (Ion Exchange Chromatography) eluate; 3: GF (Gel Filtration) eluate.

With respect to rhGCase purification, the high ionic strength of the extract superimposed the choice of HIC (Hydrophobic Interaction Chromatography) for GCase capturing and IEC (Ion Exchange Chromatography) for intermediate purification. All chromatographic fractions were analysed by ELISA, SDS-PAGE and Western blotting. Under the conditions reported in this paper, both chromatographies were quite efficient in enzyme recovery as well as in the exclusion/removal of contaminants. In the final polishing step with Hiprep 16/60 Sephacryl S-100 column, the enzyme was collected in a single peak after a retention volume equal to 56.4 mL, which was very close to that of commercial imiglucerase, equal to 55.9 mL. Total removal of contaminants was confirmed in SDS-PAGE (Figure 7) and ELISA analyses based on titration curves made with commercial therapeutic-grade imiglucerase. Data regarding the efficiency of each chromatographic step are shown in Table 1.

**Table 1:** **Efficiency of chromatographic steps in rhGCase purification**

Chromatographic step	HIC	IEC	GF
GCase recover	73.3%	75.9%	74.3%
*Cumulative GCase loss*	*26.7%*	*44.4%*	*58.7%*
Removal of contaminants	98.4%	82.9%	100%
*Cumulative removal*	*98.4%*	*99.7%*	*100%*

### Seed viability

**Figure 8:**
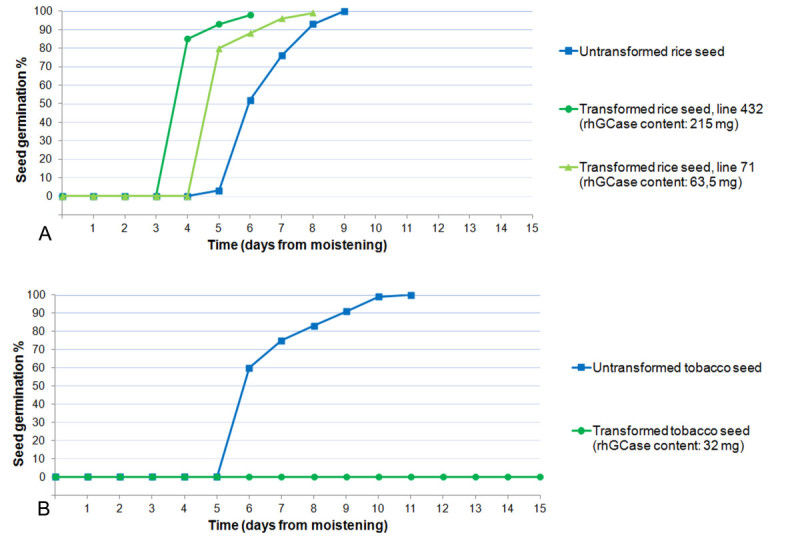
**Results of seed viability tests in rice and tobacco.** **A**: untransformed and transformed rice seeds; **B**: untransformed and transformed tobacco seeds.

Percent viability was excellent (99-100%) in both transformed seed samples and untransformed control. No dormancy was recorded in any seed lot, consistently with previous observations on germination behaviour of the CR W3 variety. As shown in Figure 8A, seed viability and the time course of germination were similar for untransformed control and two transformants with a sharply different rhGCase content (63.5 and 215 mg per kg of flour). In contrast, best tobacco transformants produced according to Reggi et al. (2005) had a seed germination capacity equal to zero (Figure 8B). It is worthy of note that the best tobacco plant had 32 mg rhGCase per kg of seed flour, i.e. approximately one half of the enzyme content recorded in the rice lower producer. This evidence demonstrates that rhGCase accumulation and seed germination can be effectively uncoupled through endosperm-specific expression in a monocot system.

### Peptide mass fingerprinting and N-terminal sequencing

**Figure 9:**
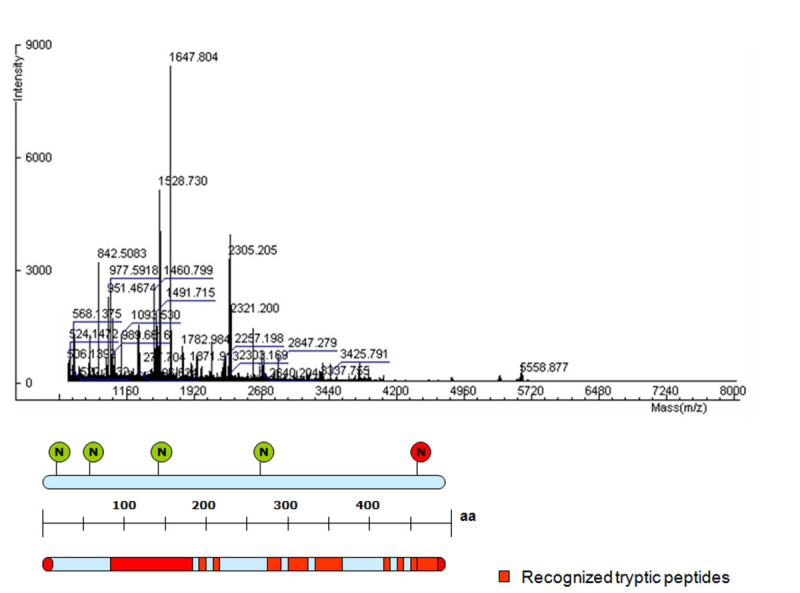
**MALDI-TOF analysis of a rhGCase sample purified by HIC and IEC chromatography.** Red bars indicate rhGCase tryptic peptides assigned to human GCase regions. The position of the five putative N-glycosylation sites is shown by circles; all sites except the last one, which never harbours glycans (Berg-Fussman et al.^1993^), are normally occupied by oligosaccharidic chains.

MALDI-TOF analysis carried out on a purified rhGCase sample digested with trypsin confirmed its equivalence with human GCase [GenBank: J03059]. The results achieved with the Mascot proteomic software (http://www.expasy.org/tools) were clear-cut: rhGCase identification was obtained with a score of 10^-32^ and the percent protein coverage was equal to 53% (Figure 9). It is interesting to note that both the amino- and the carboxy-terminus of the protein were included in the list of recognized peptides and that they resulted fully equivalent with the corresponding sequences of human GCase. Differently, the peptides containing the first, second and fourth N-glycosylation site were not detected through the Mascot analysis, because in all likelihood these sites were occupied by oligosaccharidic chains. More details on glycan structure are reported hereinafter.

The identity of the rhGCase N-terminal sequence was also determined through conventional Edman analysis. The resulting data confirmed that the first 9 amino acids of rhGCase correspond exactly to the first tract of the human mature polypeptide. Hence, it was demonstrated again that the signal peptide of glutelin 4 is well recognized and properly removed from the precursor protein during traslocation in the ER.

### Glycan structure analyses

**Figure 10:**
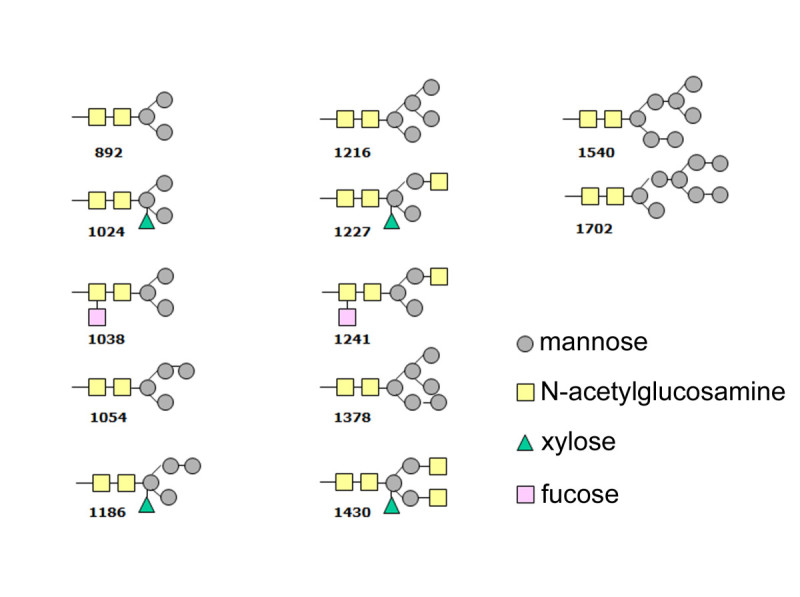
**N-glycan structures and related molecular masses predicted in rice-derived rhGCase by Glycomode analysis.**

N-glycosylation was partially characterized by digesting a purified rhGCase sample with PNGase F. MALDI-TOF data processing of the oligosaccharidic chains suggested the presence of 12 glycan forms. Figure 10 reports the glycan structures identified in rhGCase with the Glycomode software tool (http://www.expasy.org/tools). All oligosaccharide chains shared the typical paucimannosidic structure of N-linked glycans of PSV proteins (Lerouge et al.^1998^). In all cases but one, they were found to have at least one chain-terminating mannose residue needed for GCase uptake by human cells. Even though MALDI-TOF analysis does not allow a precise quantification of the different forms, mass spectra showed that Man3GlcNAc2 with or without beta(1,2)-xylose and alpha(1,3)-fucose is most abundant. Differently, glycoforms with a mass greater than 1.3 kDa were detected at much lower levels.

### Enzyme activity

**Figure 11:**
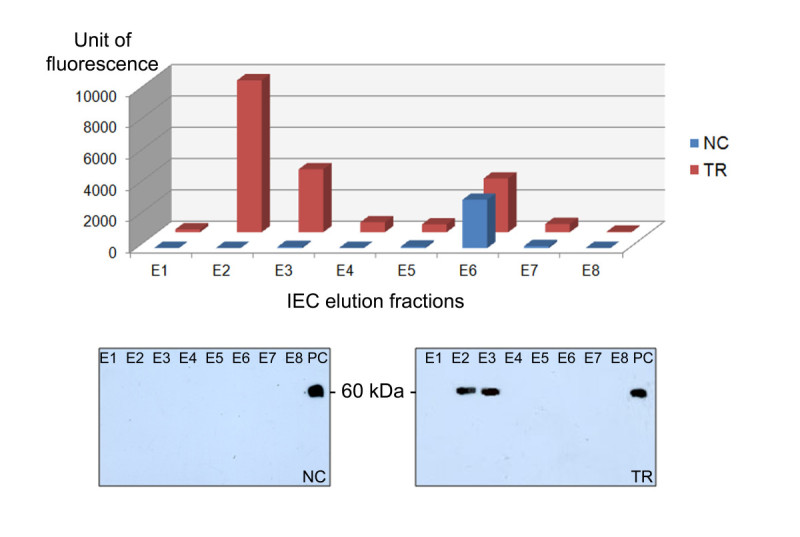
**Fluorescence recorded in comparative 4-MUG catalytic assays performed on 8 IEC elution fractions.** NC (negative control): untransformed CR W3; TR: transformed CR W3 plants; E1-E8: elution fractions; PC (positive control): 100 ng imiglucerase.

Similarly to what was found in tobacco leaf and seed extracts (Reggi et al.^2005^), a straightforward determination of rhGCase content in polished seed by enzymatic assay was prevented by the presence of an intrinsic GCase-like activity. No investigation was made on the enzyme(s) responsible for that, essentially because this endogenous component was completely separated from rhGCase during IEC (Figure 11). Hence, enzymatic assays were conducted only on purified rhGCase, using commercial imiglucerase as positive control. In particular, 5 ng of the commercial standard showed an average specific activity of 9.2 μmol/min/mg; equal amounts of rhGCase from three independent lots were repeatedly found in the range of 7.9-9.7 μmol/min/mg.

## Discussion

Among different methods described for the production of recombinant proteins in plants, those involving the seed are particularly attractive because seeds are natural organs devoted to the production and accumulation of storage proteins in a small volume and in a stable biochemical environment. In particular, neo-synthesized proteins are targeted to subcellular storage compartments such as protein bodies and protein storage vacuoles, where they can be preserved for a long time due to a low water content and the presence of proteinase inhibitors (Takaiwa et al. 2007).

In former experiments (Reggi et al. 2005), it was demonstrated that recombinant human GCase can be stored in a stable and active form in tobacco seed. However, in the best primary transformants, seed viability appeared totally impaired and all attempts to overcome the problem were unsuccessful (Reggi et al.^2005^). This evidence, together with a high oil content and tiny dimension of tobacco seed, prompted us to consider rice as an alternative host-species.

In seed-specific expression studies carried out in tobacco, recombinant proteins are typically accumulated in the cotyledon parenchymatic tissue, hence they can directly interfere with embryo development and seed germination. Differently, in rice there is the opportunity to allocate recombinant proteins in the endosperm, i.e. a tissue separated by the embryo and metabolically inert at maturity. Beside this, rice seeds can be industrially processed to separate the hulls, the germ and the aleuronic layer. The removal of these components, which is relatively inexpensive, greatly favours the extraction and purification of heterologous proteins because both the lipid content and the number of protein contaminants are minimized.

Among other advantages in using rice as bioreactor, there is the availability of some non-food varieties with easily-recognizable genetic markers (Takaiwa et al. 2007). In this work, essentially for biosafety issues, the japonica waxy rice CR W3 was used; CR W3 is a registered variety with round-shaped seed developed by the Rice Research Centre of Ente Nazionale Risi (Milan, Italy) but insofar awaiting commercial exploitation. Due to an almost void amylose content, CR W3 seed can be used for the production of starch and related byproducts, whereas its employment as human food is difficult for the remarkable loss of consistency with boiling.

The endosperm-specific accumulation of rhGCase in CR W3 seed was gained through the use of the rice glutelin 4 promoter (GluB4pro). In a former study carried out with different seed-specific promoters (Qu and Takaiwa 2004), GluB4pro determined the highest level of GUS expression in transgenic rice. The maximum accumulation of the GUS protein was observed in the inner starchy endosperm, close to the late maturation stage.

In our experiments, GluB4pro showed a similar expression pattern: in fact, rhGCase was detected exclusively in the seed endosperm and not in other vegetative or reproductive tissues; moreover, SDS-PAGE and Western blotting analyses indicated that only negligible amounts of rhGCase are lost with seed polishing, a process that eliminates the germ and the aleuronic/sub-aleuronic layers (Figure 6).

Much interestingly, neither seed viability nor dormancy were affected by rhGCase endosperm-specific expression; in all instances percent viability was excellent (99-100%) and resembled that of untransformed control. These data clearly demonstrate that the problem of seed viability loss recorded for tobacco plants (Reggi et al.^2005^) is non-existent in rice.

It is important to underline that, despite seed protein content is significantly lower in rice compared to tobacco, in rice endosperm rhGCase expression levels were found much greater than those recorded in the best tobacco transformants, perhaps due to more permissive physiological conditions and the triploid nature of the tissue. The achievement of high transgene expression levels is crucial for developing a technological platform for industrial scale-up; beside promoters, several molecular elements must be considered, including 5’ UTR and the signal peptide sequence for specific subcellular localization. For the purpose of this study, the GluB4 native leader was replaced with the artificial leader LLTCK (De Amicis et al.^2007^) mainly characterized by the combination of a poly(CAA) region with a CT-rich motif. In previous studies, the LLTCK leader determined a 12.5-fold enhancement of *uid* A expression in tobacco leaf as compared with the native CaMV 35S leader sequence (De Amicis et al.^2007^). No experimental evidence about the utility of LLTCK in monocot plants has been published so far; it should be noted however that, although not in association between each other, CT- and GA-rich regions occur in the proximity of the transcription start site of many plant genes, particularly in monocots (Chen et al.^1996^).

As far as subcellular targeting is concerned, its influence on folding, assembling, post-translational modifications and stability of a protein is widely recognized (Twyman et al.^2003^; Doran^2006^; Benchabane et al.^2008^). In this study, the signal peptide of glutelin 4 (SPGluB4) was used; immunolabelling results demonstrated that this SP determined a rhGCase accumulation in the same storage compartment of GluB4, i.e. the protein storage vacuoles (PSVs) (Takaiwa et al.^1999^; Kawakatsu et al.^2008^).

Similarly to other PSV-allocated recombinant proteins (Humphrey et al.^2002^; Arcalis et al.^2004^; Vitale and Pedrazzini^2005^), both rhGCase content and activity were maintained unaltered after 12 months of storage (data not shown).

The use of a signal peptide for ER-targeting is an acquainted technology; a more challenging aspect is represented by the prediction of both SP removal and cleavage site. If the SP is not separated from the protein, the precursor-complex can remain anchored to the ER outer membrane (Yan et al.^1997^) and in any case it does not assume the correct tertiary structure. On the other hand, if the SP is removed but cleavage does not occur in the exact position, different non-authentic versions of the mature protein may arise. This condition is absolutely detrimental for therapeutic proteins since mutated forms are potentially immunogenic or provided with different pharmacodynamic properties. Some software tools were devised for prediction of the SP cleavage site in different host systems (Emanuelsson et al.^2007^). In the present work, both SignalP 3.0 (http://www.cbs.dtu.dk/services/SignalP) and SIG-PRED (http://bmbpcu36.leeds.ac.uk/prot_analysis/Signal.html) were preliminary used to determine the probability of proteolytic cleavage of SPGluB4/GCase complex; in either case, the predicted site corresponded exactly to the boundary between SPGluB4 and mature GCase (MATIAFSRLSIYFCVLLLCHGSMA//ARPCIPKSF…). In fact, N-terminal sequencing revealed that SPGluB4 is well recognized and correctly removed, thus originating a mature protein whose amino acid sequence is perfectly identical to the human one.

Another important issue for injectable proteins produced in plants concerns the type and level of N-glycosylation. Glycan analyses performed by MALDI-TOF on oligosaccharide chains of rice-derived GCase revealed that 11 out of 12 predicted glycoforms are mannose-ending, which is an essential pre-requisite for human cell internalization. This represents a further advantage in the clinical use of rice-derived GCase in comparison with CHO-derived molecule. In fact, the latter must be partially deglycosylated in order to expose mannose residues which are specifically recognized by endocytic carbohydrate receptors on macrophages of reticulo-endothelial system. This enzymatic process complicates the purification step and increases production costs (Futerman et al.^2004^). Preliminary assays conducted on human target cells demonstrated that the rice seed-derived enzyme is well-recognized and taken up at levels comparable to those of the commercial imiglucerase (data not shown).

## Conclusions

This paper describes an industrially-scalable method to produce human acid beta-glucosidase in rice endosperm. Likewise, the same procedure can be applied to efficiently produce pharmaceutical compounds which, similarly to rhGCase, are potentially harmful to the plant cell.

The platform overcomes some important difficulties faced in previous work, more specifically:

- it allows a tissue-specific expression and a consistent subcellular localization of human acid beta-glucosidase inside the rice seed;
- it allows the production of a pharmaceutical molecule suitable for therapeutic use;
- it does not affect seed viability thus enabling the maintenance and multiplication of productive lines through sexual reproduction;
- it facilitates both enzyme extraction and purification, improving the overall management of the productive process;
- it is economically more convenient compared to the CHO-technology, thus setting the conditions for a more affordable therapy of Gaucher disease.

Rice-derived rhGCase displays all the molecular characteristics and pharmaceutical prerequisites for ERT of Gaucher disease, being highly equivalent to the native human enzyme.

## M ethods

### Construction of the plant expression vector pTRS0_GCase

This vector was developed from the commercial plasmid pCAMBIA1300 [GenBank: AF234296] by introduction of the GCase cassette into the *Eco* RI site. The GCase cassette was designed to harbour the rice storage protein glutelin 4 promoter (GluB4pro), the human GCase CDS and the nopaline synthase terminator of *Agrobacterium tumefaciens* (NOS-ter).

The GluB4pro sequence was PCR-isolated from rice genomic DNA (*Oryza sativa* ssp. japonica, var. CR W3) with primers designed on the basis of GenBank acc. No. AY427571 (forward 5’- GCATGCGAATTCTACAGGGTTCCTTGCGTG-3’; reverse 5’-TCTAGAAGCTATTTGAGGATGTTATTGGAA-3’). In order to improve rhGCase expression levels, the native 5’-UTR was substituted with the LLTCK leader (De Amicis et al.^2007^). RhGCase targeting to the protein storage vacuoles (PSVs) was attempted with the transit peptide of rice glutelin 4 (Uniprot code P14614 [1–24]). Promoter, LLTCK leader and transit peptide sequences were ligated immediately upstream the GCase mature polypeptide CDS [GenBank: M16328, nt 553–2043]; the GCase coding sequence was obtained by RT-PCR from human placental total RNA. The expression cassette was completed by the addition of nos-ter.

**Figure 12:**
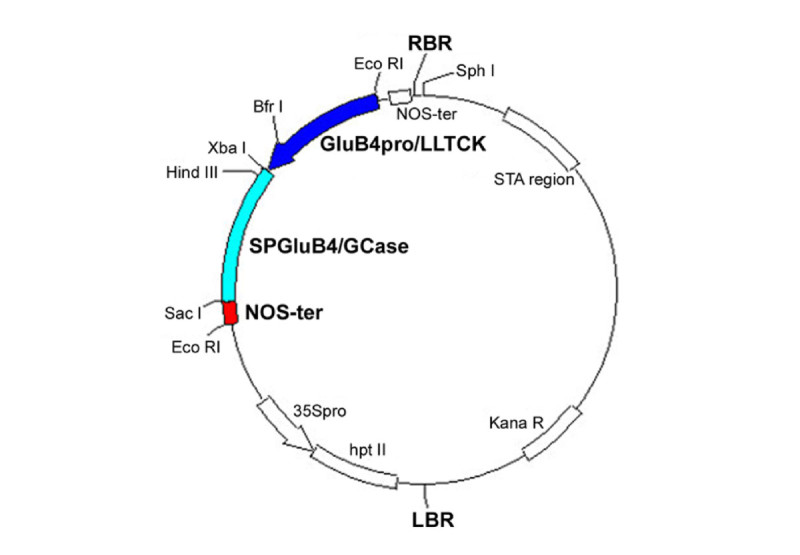
**Scheme of the pTRS0_GCase vector.** The rhGCase cassette and the restriction sites used for sequence assembly are highlighted. Abbreviations: Glub4pro/LLTCK = rice glutelin 4 promoter with LLTCK 5’ UTR; SPGluB4/GCase = *hGCase* gene with the signal peptide of rice glutelin 4; NOS-ter = nopaline synthase terminator.

After cloning into the final expression vector pTRS0_GCase (Figure 12), all sequences were verified on both strands.

### Rice transformation and molecular analyses on primary transformants

The engineered vector was introduced into *Agrobacterium tumefaciens*, strain EHA105, by electroporation. Rice transformation was carried out according to Hiei et al. (1994) with minor modifications. Putatively transformed (i.e. hygromycin resistant) plants were potted in peat, hardened in a greenhouse and maintained until seed maturation. For PCR analyses, genomic DNA was extracted from young leaves according to Doyle and Doyle (1990) and analysed with two primer pairs (forward1 5’-TCTAGAATGGCCACCATTGC-3’ and reverse1 5’-GAGCTCTCACTGGCGATGCC-3’; forward2 5’-AATAGCTGCGCCGATGGTTT-3’ and reverse2 5’-ACCACGGCCTCCAGAAGAAG-3’) with the following PCR conditions: one cycle of 5 min at 95°C, 40 cycles of 40 s at 95°C, 40 s at 58°C and 2 min at 72°C, followed by a final extension step of 5 min at 72°C.

In order to estimate GCase copy number in transgenic rice plants, we used the method of real-time quantitative PCR developed by Ding et al. (2004) in which the rice sucrose phosphate synthase (*SPS*) gene represents the endogenous reference standard. The oligonucleotide primers and TaqMan probes (Table 2) were designed with the Primer 3 Output software (http://frodo.wi.mit.edu). All probes had the Blackhole Quencher 1 at the 3’ end, whereas in 5’ they were labelled with the fluorescent reporter dye Texas Red (SPS-P) or Joe (GCase-P).

**Table 2:** **Primer pairs and probes used in real**-**time quantitative PCR**

Name	Sequence (5’-3’)
SPS for	AACGGATATCTTTCAGTTTGTAACCAC
SPS rev	CGGTTGATCTTTTCGGGATG
SPS-P	GATGACGCACGGACGGCTCG
GCase for	CCTGCCCTTGGTACCTTCAG
GCase rev	GCCCCATACTCAGCTCCATC
GCase-P	GAGAGTACACGCAGTGGGCGACG

RT-PCR was performed with the thermal cycler iCycler iQ real-time PCR detection system (Bio-Rad) and 1xPlatinum Quantitative PCR SuperMix UDG (Invitrogen). The amplification conditions consisted of one cycle of 3 min at 50°C and 3 min at 95°C, followed by 50 cycles of 15 s at 95°C and 1 min at 60°C. In order to generate standard curves for DNA quantification, SPS- and GCase-harbouring plasmids were serially diluted and analyzed.

### Protein extraction

Protein extracts for Western blot analyses were prepared as follows: five dehulled seeds were ground with 1 mL of extraction buffer (500 mM NaCl in 50 mM Tris-HCl buffer, pH 7.0) using mortar and pestle. The resulting homogenate was incubated at RT for 5 min under agitation and then centrifuged at 15000 × g for 45 min at 4°C. The supernatant (30 μg soluble protein) was directly used for SDS-PAGE.

Protein extracts for ELISA analyses were obtained homogenizing five dehulled seeds with 10 mL extraction buffer, as above.

To determine rhGCase expression levels, 40 dehulled seeds were milled and thoroughly homogenized in 80 mL extraction buffer and stirred on ice for 1 h. The extract was centrifuged at 20000 × g, 4°C, for 40 min. The supernatant was collected and stored for DAS-ELISA.

### Gel electrophoresis and staining

SDS-PAGE was performed under standard conditions, using the Mini Protean II apparatus (BioRad), loading the samples in 10% polyacrylamide gels after denaturation for 5 min. Commercial molecular weight markers (Fermentas) and commercial imiglucerase were used to assess the molecular weight of rhGCase. Gels were stained with EZBlue^TM^ Gel Staining Reagent (Sigma Aldrich) or with ProteoSilver^TM^ Silver Stain kit (Sigma Aldrich).

### Western blot

The extracts prepared as above were denaturated under non-reductive conditions and loaded in a 10% polyacrylamide gel together with Precision Protein Standard (BioRad). The gel was electroblotted to a 0.2 μm PVDF membrane (Millipore) with the Trans-blot SD apparatus (BioRad). The blot was blocked with 7.5% non-fat dry milk (Oxoid) in PBS for 1 h at RT. After 3 washes with PBS Tween 0.1%, the primary rabbit polyclonal antibody, produced using commercial imiglucerase (Genzyme Corp., Sanofi Aventis) as antigen, was diluted 1:1000 in the blocking buffer and the blot was incubated for 1 h at RT. Then, the HRP-conjugated secondary antibody (Sigma Aldrich) was diluted 1:10000 and the blot incubated again for 1 h at RT. After final washes, chemiluminescence was developed with ECL plus (GE Healthcare). To identify the best transgenic lines, samples showing a strong signal were further evaluated by Western blotting using two- or four-fold diluted supernatants.

### RhGCase quantification by DAS-ELISA

A double antibody sandwich ELISA was used to quantify rhGCase in both crude protein extracts and chromatographic fractions. Capture antibody was obtained from the same rabbit antiserum used in Western analyses, purified by affinity chromatography on imiglucerase derivatized resin. Detecting antibody was produced by conjugating HRP with capture antibody. Both antibodies were purchased from Davids Biotechnologie (Germany).

SPL maxi binding plates (SPL, Korea) were coated with 2 ng/μL of capture antibody in coating solution (30 mM NaCl, 2.5 mM sodium phosphate buffer, 15 mM sodium azide, pH 7.4), 100 μL per well. Coating was performed by stirring the plate at 37°C for 20 min. Then, the plate was blocked with 3% BSA solution in PBS with 15 mM sodium azide, at 37°C for 20 min, 300 μL per well. Crude extracts were diluted 1:30 in dilution buffer (1% BSA, PBS-Tween 20 0.1% solution, pH 7.4), added to the plate and incubated at 37°C for 20 min, 100 μL per well. Chromatographic fractions were differently diluted case by case. After three washes with PBS-Tween 20 0.1%, detecting antibody was applied at 0.8 ng/μL in dilution buffer, 50 μL per well at 37°C for 20 min. After four washes, detection was performed using TMB Liquid substrate system (Sigma Aldrich), 100 μL per well; the reaction was stopped with 1 M HCl, 100 μL per well. Plates were read with Modulus II microplate reader (Turner Biosystem) at 450 nm.

For rhGCase quantification, a standard curve was built with the following commercial imiglucerase concentrations: 250, 125, 62.5, 31.3 15.6, 7.8, 3.9, 2.0 pg/μL in dilution buffer, 100 μL per well.

To evaluate rhGCase expression levels in polished seed, serial extracts were diluited 1:100 in ELISA dilution buffer and analyzed separately. The total rhGCase amount was calculated and expressed as mg per kg of flour.

### Tissue-specific expression analyses

In order to demonstrate the endosperm-specific expression of rhGCase, Western blot analyses were performed on crude protein extracts obtained from different organs/tissues at the vegetative and reproductive stage. Protein samples were taken from: leaf blade, leaf sheath, stem internode, stem node, root, rachis, glume and husk, unfertilized ovule, immature anther, mature pollen, immature embryo, endosperm, using Tris-HCl 50 mM, 0.5 M NaCl, pH 7.0 in mortar and pestle. To extract proteins, the resulting homogenates were incubated on ice for 1 h under agitation and then centrifuged at 15000 × g for 45 min at 4°C. Protein concentration of the supernatants was determined by a standard Bradford assay (Bradford^1976^). Samples (7–15 μg) were loaded in Laemmli buffer (Laemmli^1970^) into a 10% polyacrylamide gel together with a molecular weight marker (Sigma Aldrich) and a positive control (commercial imiglucerase). At the end of the run, proteins were stained overnight with Coomassie and destained with 20% methanol and 10% acetic acid. A second electrophoretic gel was used to blot the proteins onto a PVDF membrane and perform a Western blot analysis as reported in the previous paragraph. SDS-PAGE and Western blotting were performed also on samples extracted from wild type CR W3. Results achieved by Western blotting were eventually confirmed in DAS-ELISA.

### Electron and immunoelectron microscopy

Unlabeled osmium-treated samples were prepared according to Musetti et al. (2011). For immunoelectron microscopy, immature rice seeds (approx. 10–15 days after flowering) were fixed in 0.2% glutaraldehyde, rinsed in 0.1 M phosphate buffer, pH 7.4, dehydrated in ethanol (25, 50, 75%, 30 min each step, then 100% for 1 h), then infiltrated in LR White Resin (LRW, London Resin) with the following ratio: LRW:ethanol 1:2 for 30 min, LRW:ethanol 2:1 for 30 min, and 100% LRW (two changes: after 1 h, then overnight) at RT. The samples were embedded in fresh LRW resin in BEEM capsules and polymerized at 60°C for 24 h.

Ultra-thin sections (60–70 nm) were cut with the ultramicrotome LKB Nova (Reichter) and mounted on nickel grids (Electron Microscopy Sciences) for immunolocalization. Sections were incubated with Normal Goat Antiserum (Aurion) diluted 1:30 in buffer C (0.05 M Tris-HCl, pH 7.6, 0.2% BSA) for 15 min and then with the anti-GCase serum diluted 1:500 in buffer C for 1 h at RT. After washes, the sections were incubated for 1 h with a solution of goat anti-rabbit antibody conjugated with 15 nm colloidal gold (Aurion) diluted 1:40 in 0.02 M Tris-HCl, pH 8.2, containing 0.9% NaCl and 1% BSA. Sections were stained with 0.1% lead citrate (Reynolds^1963^) and examined with the CM10 transmission electron microscope (Philips).

### Seed viability

One hundred non-vernalized seeds produced by the best transgenic lines were placed onto moistened paper and incubated in petri dishes at 28/22°C d/n, 16/8 h d/n; seeds were daily controlled to determine their viability using untransformed, non-vernalized CR W3 seed as control.

### RhGCase purification

Transgenic rice seed was dehulled, polished with the TO-92 equipment (Satake) and milled in a coffee blender. Rice flour was suspended in cold methanol (1:5 w/v), stirred on ice for 5 min and centrifuged for 15 min at 14000 × g. After the supernatant was discarded, the pellet was extracted with 2 mM sodium citrate, pH 5.5 (1:5 w/v) and centrifuged for 15 min at 14000 × g; this step was done to eliminate the low-salt soluble protein fraction and for a better efficiency it was repeated twice. At the end of pretreatment, the pellet was extracted with 0.5 M NaCl in 50 mM Tris-HCl buffer, pH 7.0 (1:5 w/v). The extract was stirred for 5 min at RT and then centrifuged for 15 min at 14000 × g to recover the supernatant. In order to extract all the rhGCase protein stored in rice seed, the extraction procedure was repeated three times and the supernatants were pooled and further clarified by centrifugation at 14000 × g for 45 min at 4°C.

RhGCase was purified using a three-step purification process involving HIC (Hydrophobic Interaction Chromatography) for capture, IEC (Ion Exchange Chromatography) for intermediate purification and GF (Gel Filtration) for the polishing step. All chromatographies were performed with the AKTA Prime purification system (GE Healthcare).

For HIC, two 5-mL HiTrap Octyl FF columns (GE Healthcare) were serially connected and equilibrated with one column volume of loading buffer (10 mL of 200 mM ammonium sulphate in 50 mM Tris-HCl buffer, pH 7.0). A 3 M ammonium sulphate solution was added to the clarified extract to reach a final concentration of 200 mM ammonium sulphate. After filtration with 0.2 μm filter (Millipore), 300 mL of the extract were applied to the column at a 5 mL/min flow-rate. The flow-through was reloaded onto the column until three loading volumes were passed. The flow-through was then collected and stored until analysis. The column was washed with 14 column volumes (140 mL) of 15 mM sodium citrate buffer, pH 5.5. Elution was carried out by increasing ethylene glycol concentrations from 0% to 50% in 15 mM sodium citrate buffer, pH 5.5. One-mL fractions were collected and tested in ELISA. Positive fractions (from number 3 to number 15) were pooled and loaded in a 1-mL HiTrap SP HR column (GE Healthcare); rhGCase was eluted in 5-mL fractions by a continuous gradient from 60 mM to 200 mM of NaCl in 20 column volumes (20 mL).

Gel filtration was used as the final polishing step. A HiPrep 16/60 Sephacryl S-100 High Resolution column (GE Healthcare) was equilibrated with 15 mM sodium citrate and 150 mM NaCl, pH 5.5; the column was then loaded with the DAS-ELISA positive IEC fraction (number 3), reduced to 500 μL by Amicon Ultra-15 (MWCO 30 kDa, Millipore) and run at a flow-rate of 0.5 mL/min. In order to recognize the rhGCase-containing peak, a preliminary GF was performed with commercial imiglucerase.

### Enzymatic assay

RhGCase activity was assayed using 4-MUG (4-methylumbelliferyl β-D-glucoside, Sigma Aldrich) as substrate. The reaction buffer contained 100 mM sodium acetate buffer, pH 5.5, 0.5% w/v taurocholate and 10 mM 4-MUG, 50 μL per well. The reaction was carried out at 37°C for 30 min and finally stopped with 200 μL 100 mM glycine-NaOH, pH 10.0. Fluorescence was read at 410–460 nm with the fluorimeter Modulus II microplate reader (UV Optical kit, Turner Biosystem) following excitation at 365 nm. A standard curve was built with 2.5-5.0 nmol 4-MU (4-methylumbelliferone, Sigma Aldrich) in reaction buffer, 200 μL per well.

### Peptide mass fingerprinting and N-terminal sequencing

Peptide mass fingerprinting was performed by MALDI-TOF technology (Perkin Elmer). A purified rhGCase sample was electrophoresed and stained with Coomassie Blue R-250 staining; the band of interest was cut, washed at 37°C with a 100 mM NH_4_HCO_3_ and acetonitrile (ACN) (50:50 v/v) solution, ground and dehydrated with ACN. The protein was reduced with 10 mM dithiothreitol, alkylated with 50 mM iodoacetamide (IAA) and overnight digested with 50 ng/μL trypsin (Promega) at 37°C. After a further treatment with 2% formic acid and 60% ACN, peptides were purified with a zip-tip C18 (Millipore), removing the contaminants with 0.1% TFA washes. Peptides were slowly eluted with ACN and 0.1% TFA (70:50), mixed with a CHCA (α-cyano-4-hydroxycinnamic acid) matrix and spotted onto a MALDI plate for analysis.

For N-terminal sequencing, a purified rhGCase sample was electrophoresed and blotted onto a PVDF membrane. The rhGCase band was identified by Coomassie-Blue staining. The N-terminal sequence was determined by Primm Srl (Milan, Italy) using the Edman degradation method (Edman^1950^).

### Glycan structure analyses

Glycosylation patterns were determined using exoglycosidase digestion and MALDI-TOF analyses. In particular, a purified rhGCase sample was run on SDS-PAGE and the band of interest, identified by Coomassie-Blue staining, was cut out and incubated with PNGase F, i.e. peptide-N(4)-(N-acetyl-β-glucosaminyl) asparagine amidase F, to remove the oligosaccharide chains connected to the amidic group of asparagine residues. Oligosaccharide chains were then analysed by MALDI-TOF according to Siemiatkoski et al. (2006).

## Authors’ original submitted files for images

Below are the links to the authors’ original submitted files for images. Authors’ original file for figure 1 Authors’ original file for figure 2 Authors’ original file for figure 3 Authors’ original file for figure 4 Authors’ original file for figure 5 Authors’ original file for figure 6 Authors’ original file for figure 7 Authors’ original file for figure 8 Authors’ original file for figure 9 Authors’ original file for figure 10 Authors’ original file for figure 11 Authors’ original file for figure 12

## Abbreviations

hGCase: human acid-beta-glucosidase
rhGCase: recombinant human acid-beta-glucosidase
GD: gaucher disease
ERT: enzyme replacement therapy
SPS: sucrose phosphate synthase
PSVs: protein storage vacuoles
HIC: hydrophobic interaction chromatography
IEC: ion exchange chromatography
GF: gel filtration
PNGase F: peptide-N(4)-(N-acetyl-beta-D-glucosaminyl)asparagine amidase F
GluB4: glutelin 4
GluB4pro: glutelin 4 promoter
SPGluB4: signal peptide of glutelin 4.
