# Supplementary figures and images for: Endosperm-specific expression of human acid beta-glucosidase in a waxy rice
Source: Rice (N Y). 2012 Dec 6;5:34. doi: 10.1186/1939-8433-5-34 (PMC4883710; doi:10.1186/1939-8433-5-34)

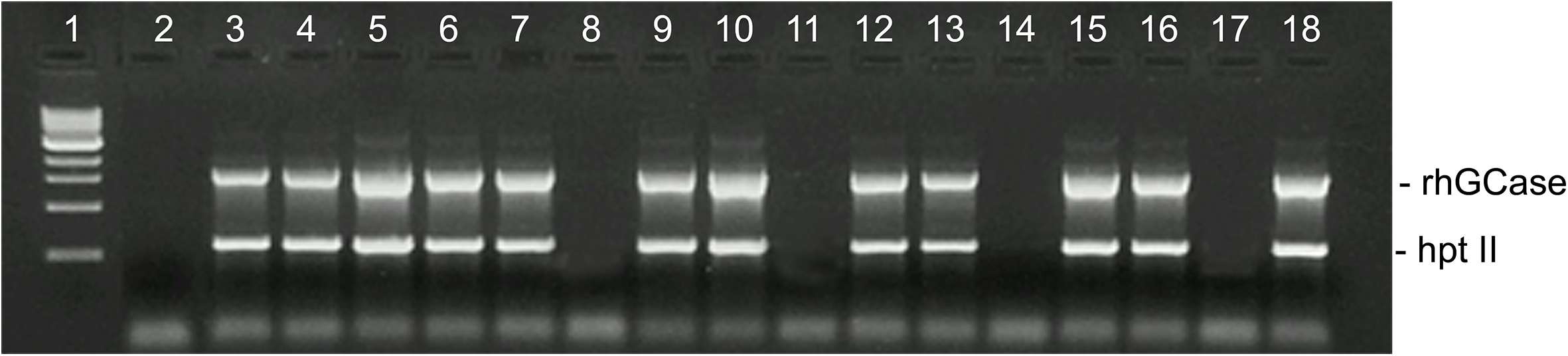

Supplement: Supplementary file 1 — Authors’ original file for figure 1 [file 12284_2012_37_MOESM1_ESM.tiff]

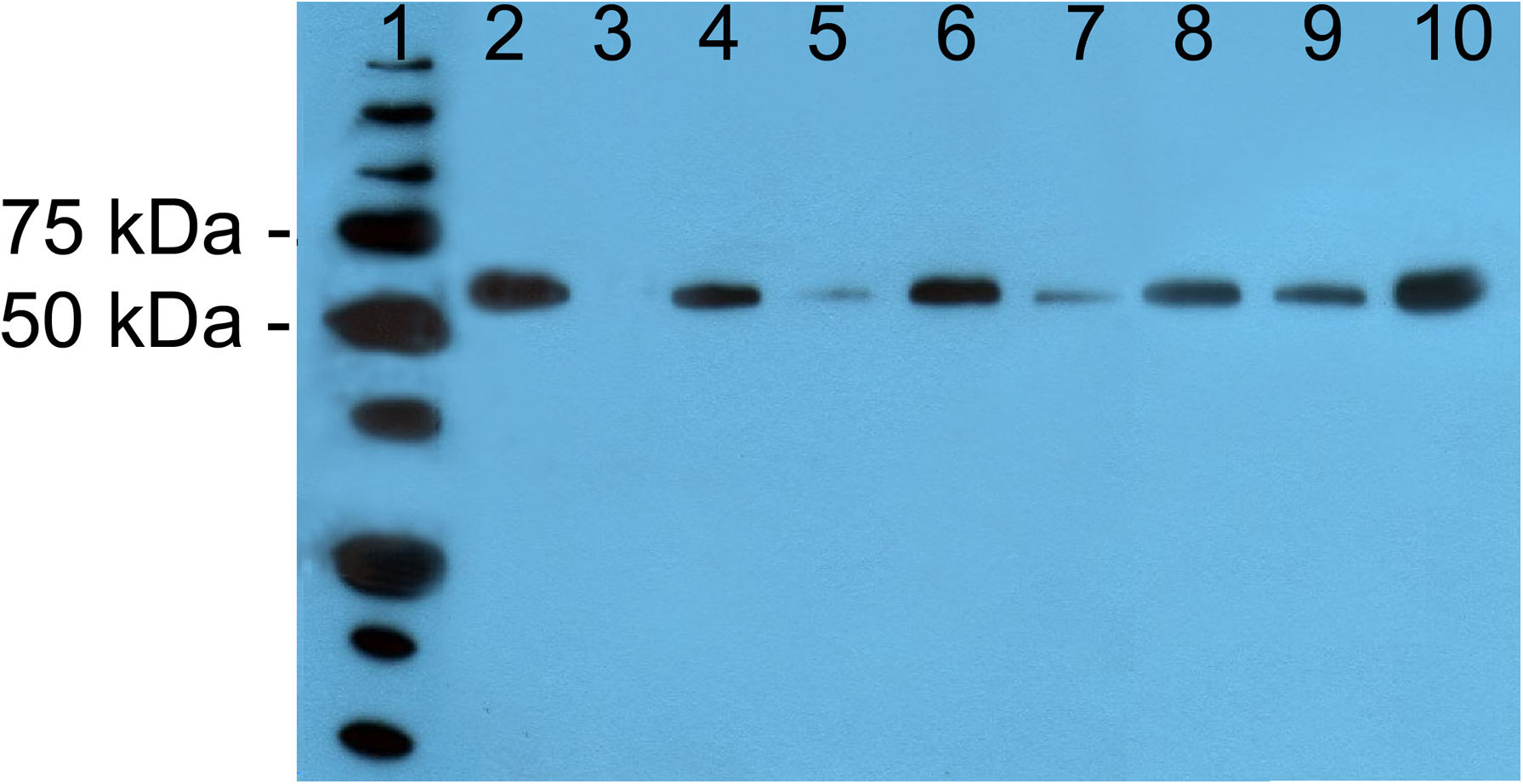

Supplement: Supplementary file 2 — Authors’ original file for figure 2 [file 12284_2012_37_MOESM2_ESM.tiff]

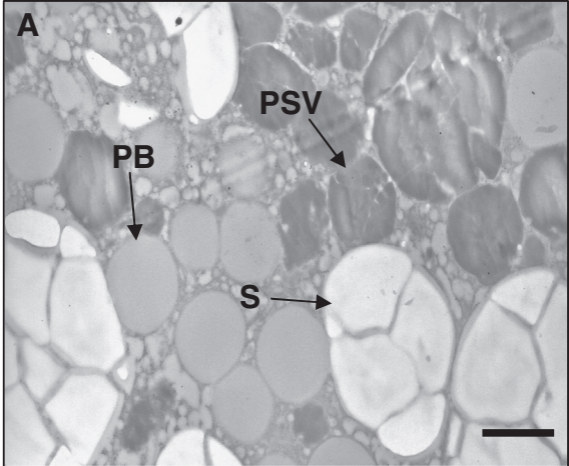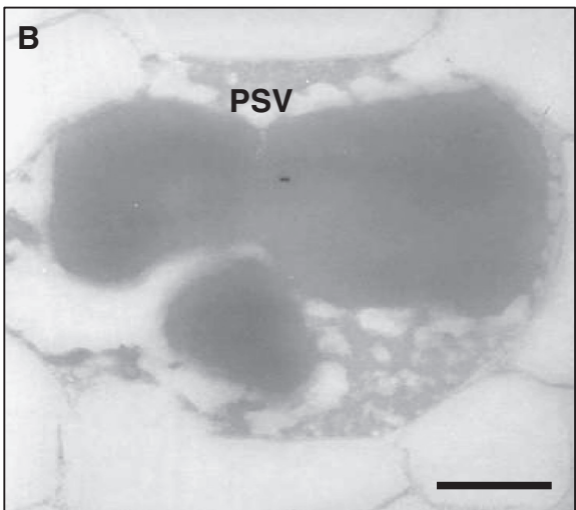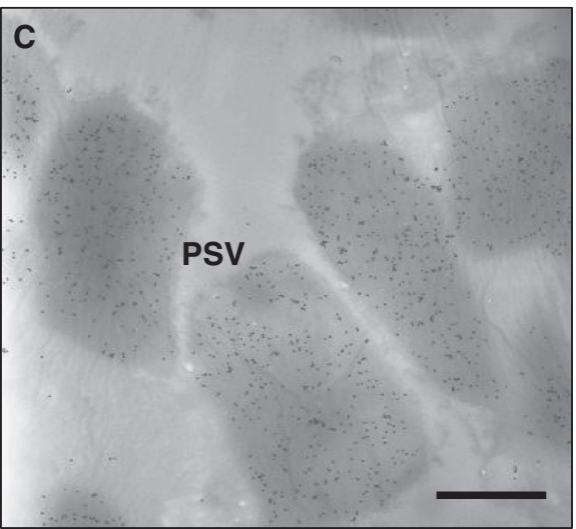

Supplement: Supplementary file 3 — Authors’ original file for figure 3 [file 12284_2012_37_MOESM3_ESM.pdf]

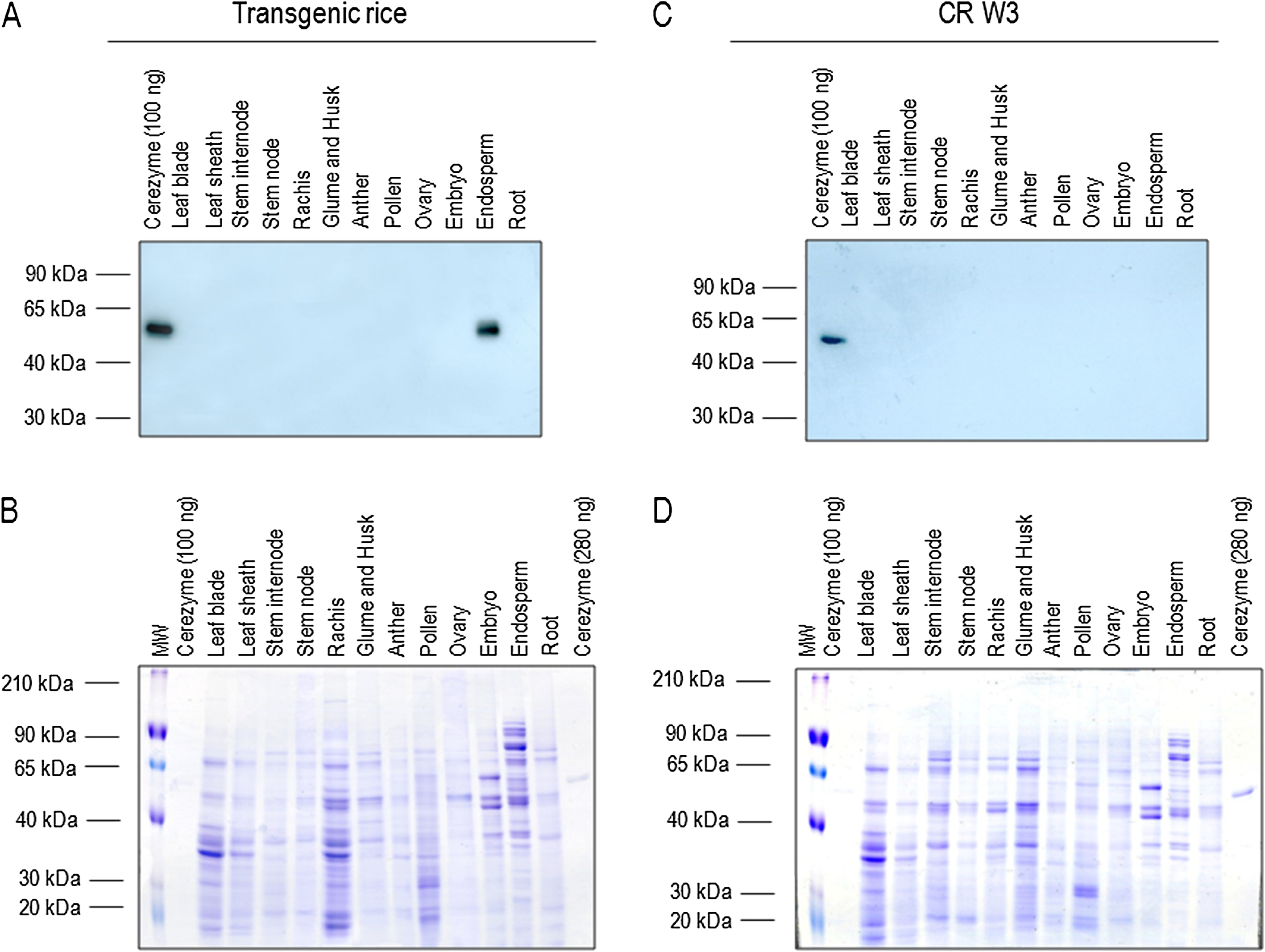

Supplement: Supplementary file 4 — Authors’ original file for figure 4 [file 12284_2012_37_MOESM4_ESM.tiff]

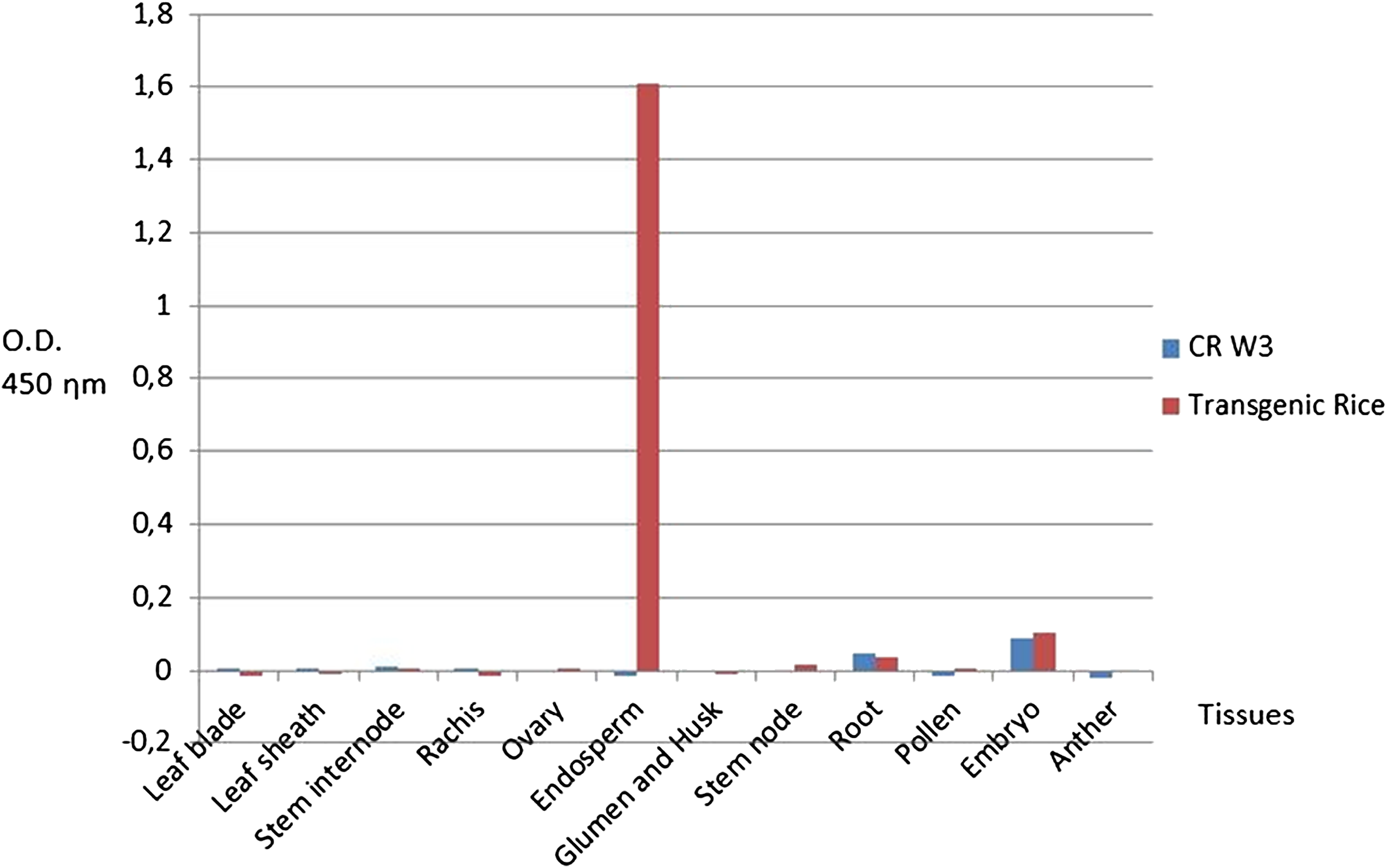

Supplement: Supplementary file 5 — Authors’ original file for figure 5 [file 12284_2012_37_MOESM5_ESM.tiff]

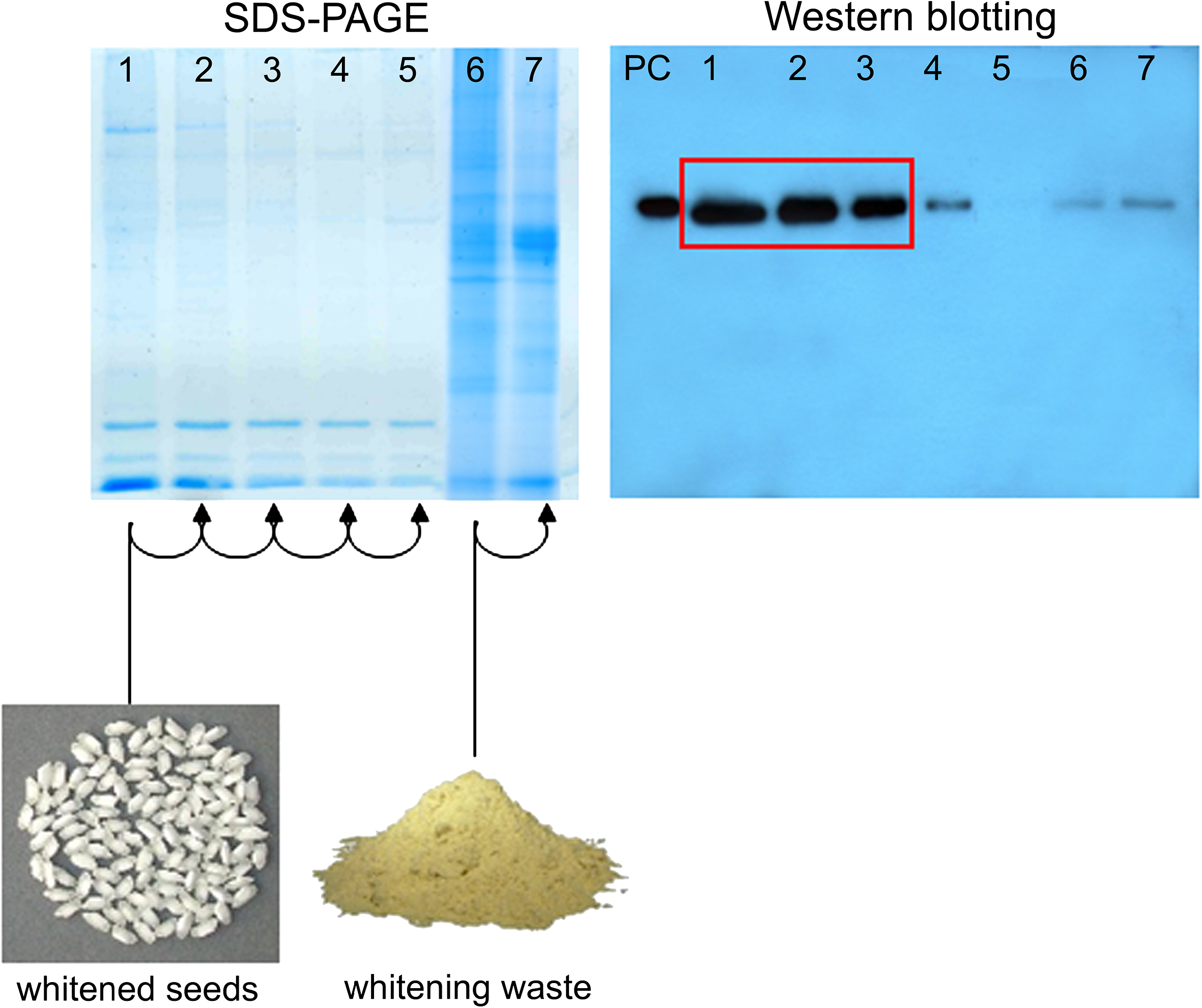

Supplement: Supplementary file 6 — Authors’ original file for figure 6 [file 12284_2012_37_MOESM6_ESM.tiff]

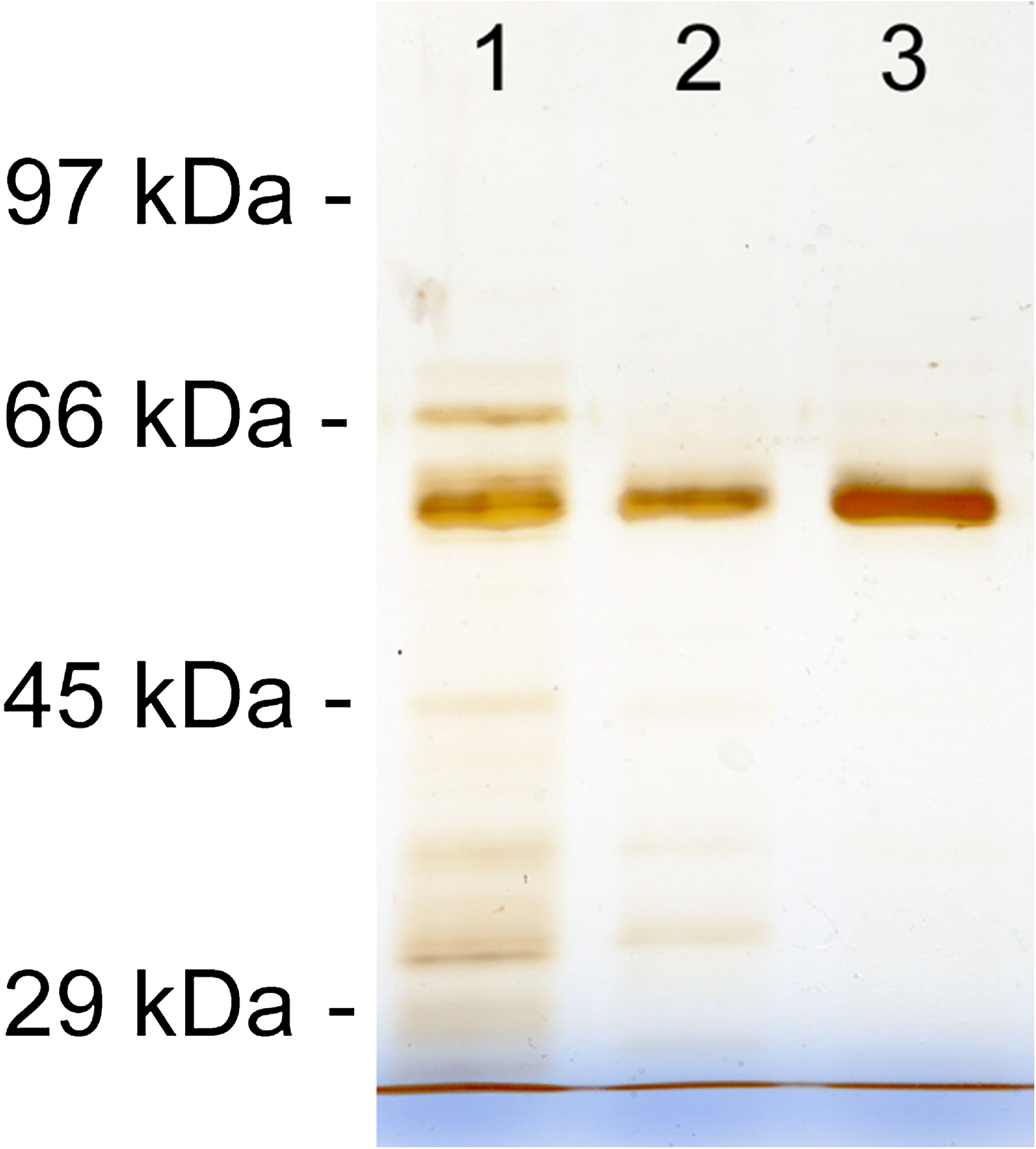

Supplement: Supplementary file 7 — Authors’ original file for figure 7 [file 12284_2012_37_MOESM7_ESM.tiff]

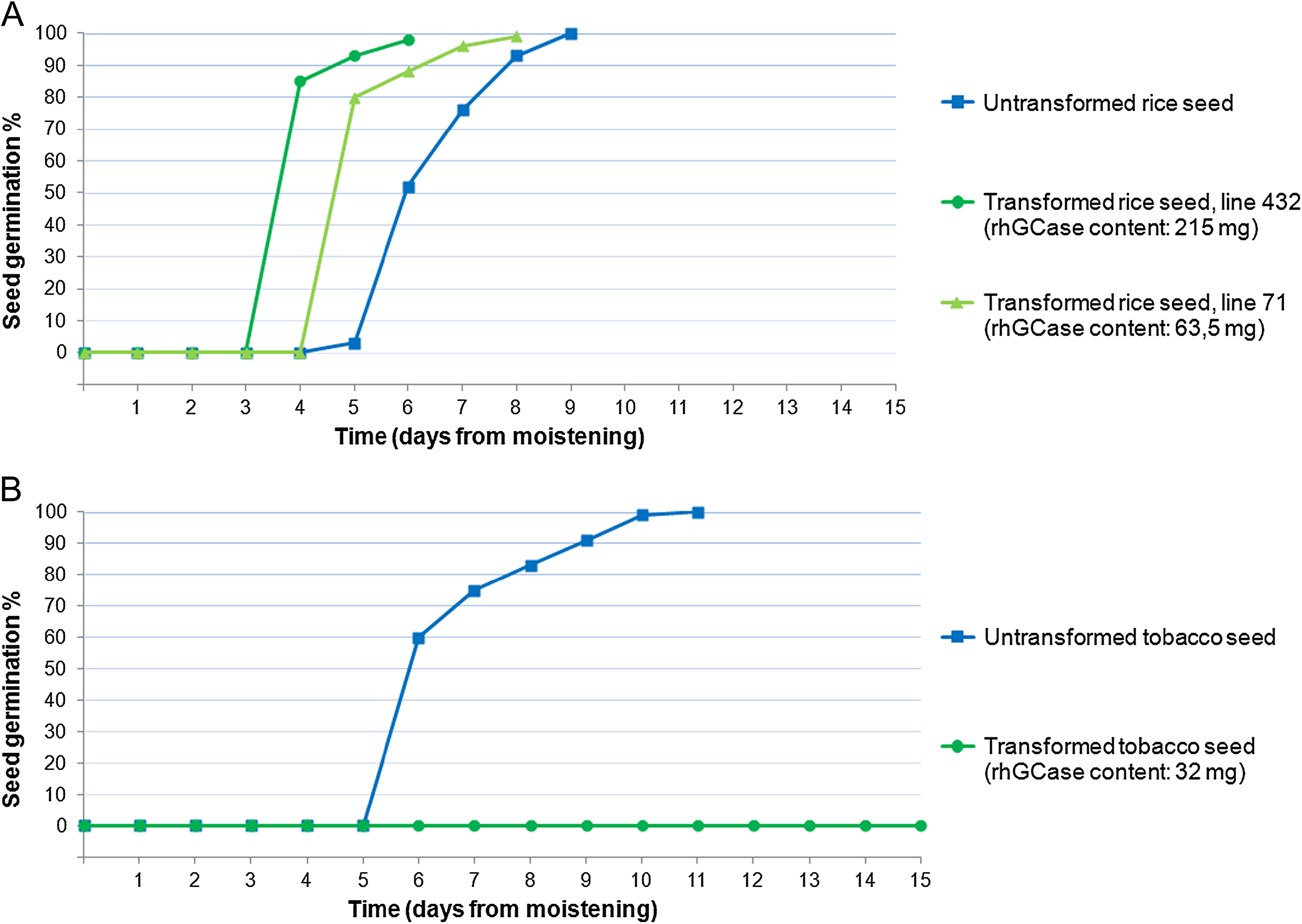

Supplement: Supplementary file 8 — Authors’ original file for figure 8 [file 12284_2012_37_MOESM8_ESM.tiff]

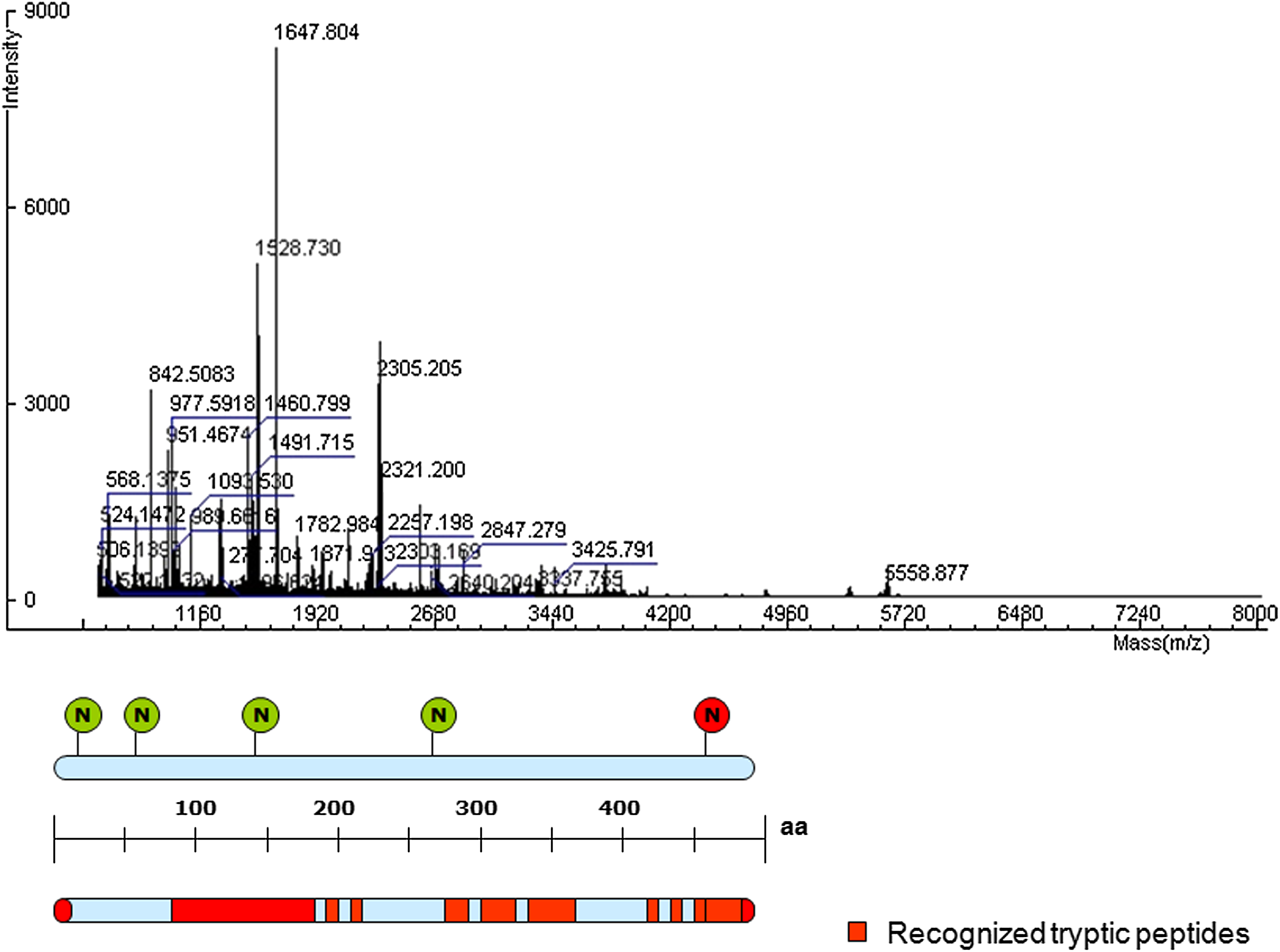

Supplement: Supplementary file 9 — Authors’ original file for figure 9 [file 12284_2012_37_MOESM9_ESM.tiff]

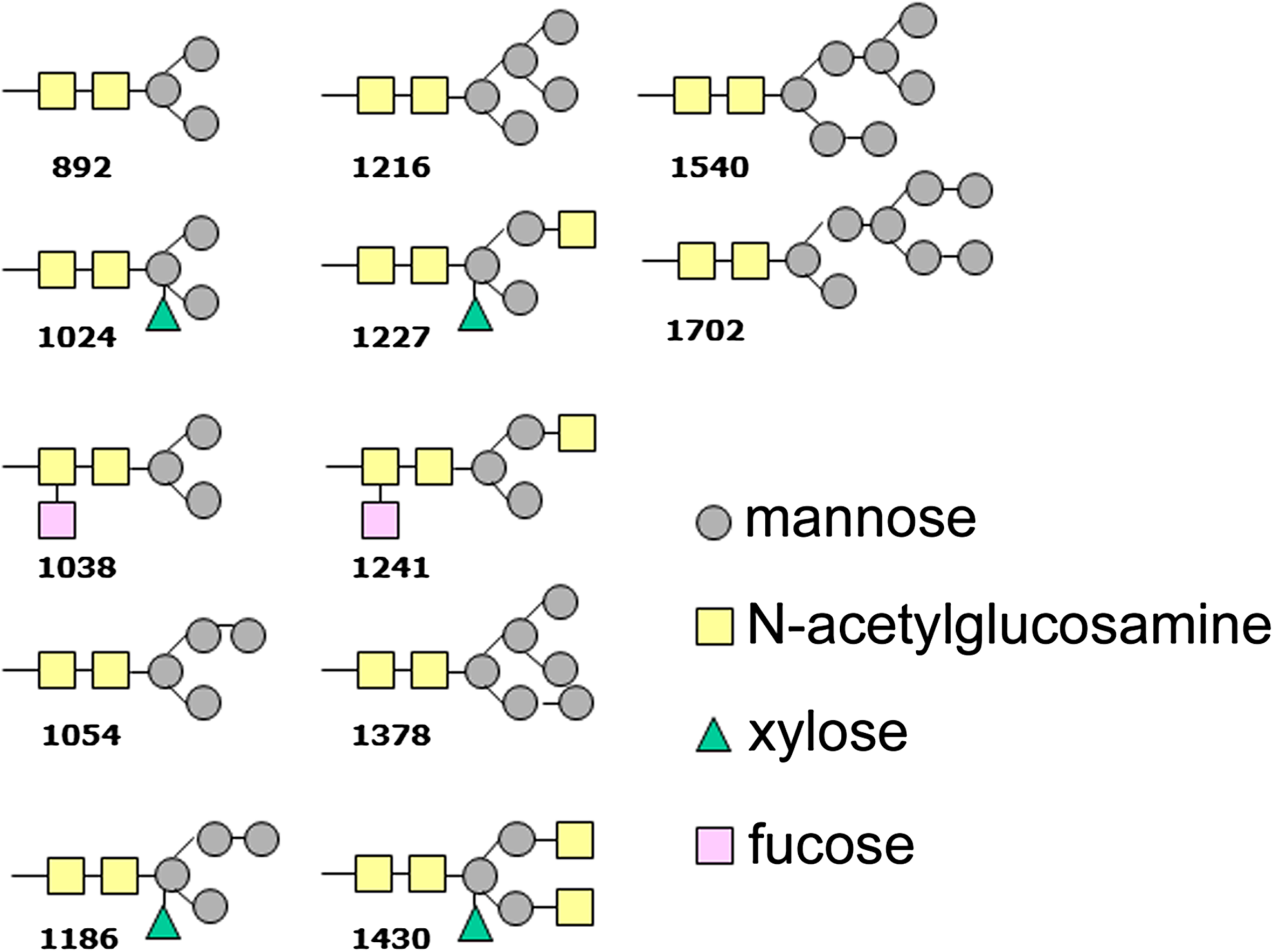

Supplement: Supplementary file 10 — Authors’ original file for figure 10 [file 12284_2012_37_MOESM10_ESM.tiff]

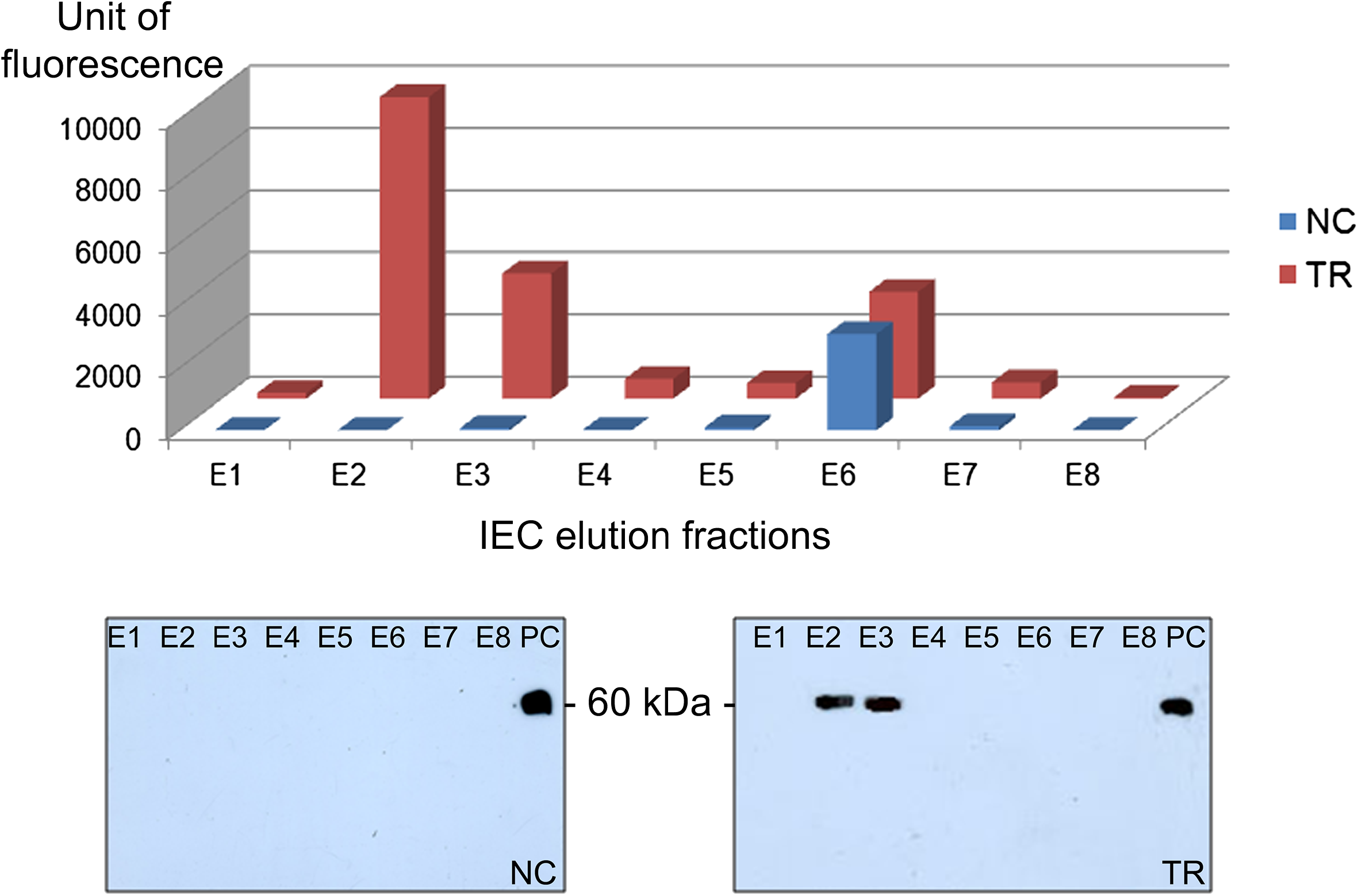

Supplement: Supplementary file 11 — Authors’ original file for figure 11 [file 12284_2012_37_MOESM11_ESM.tiff]

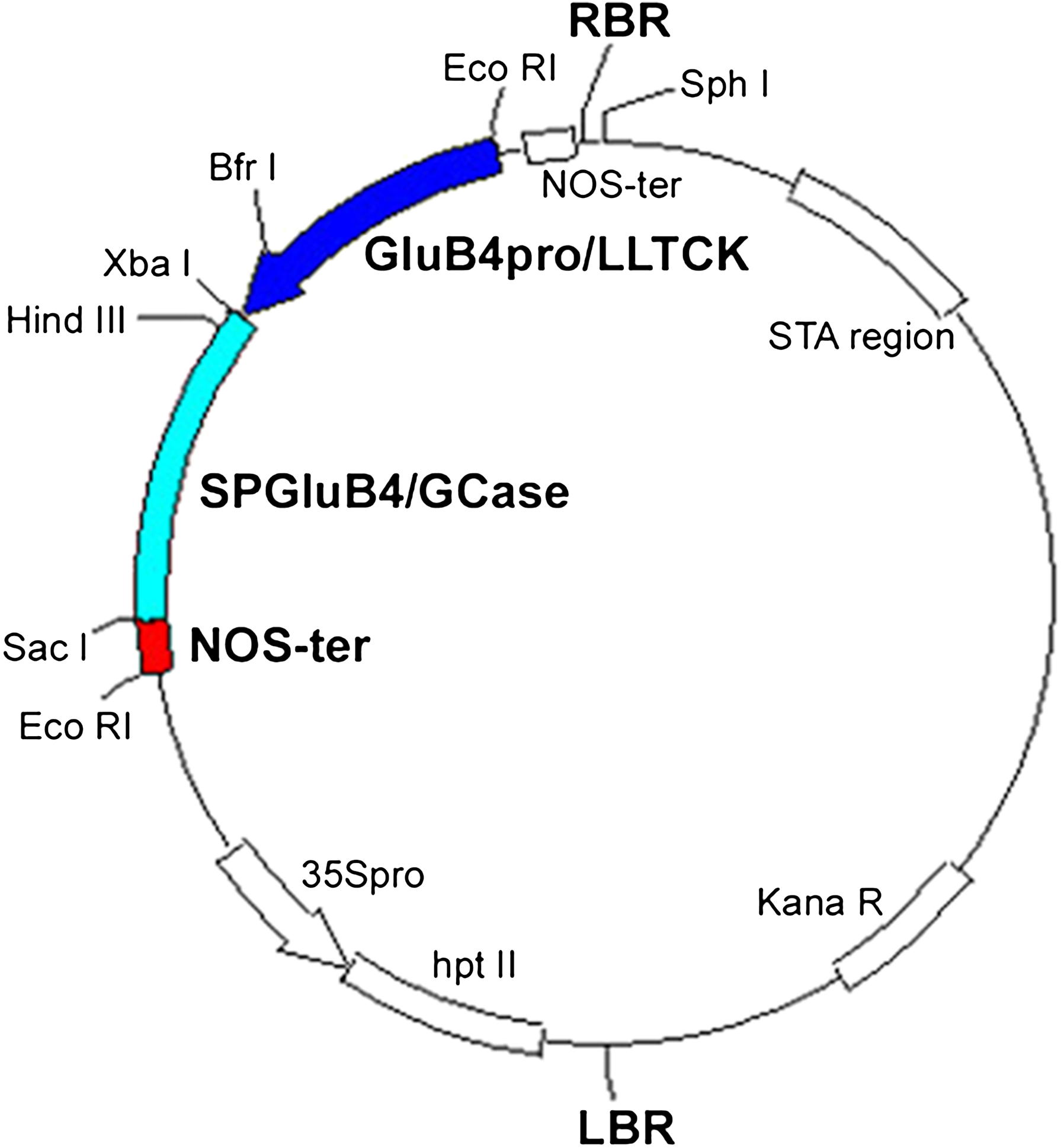

Supplement: Supplementary file 12 — Authors’ original file for figure 12 [file 12284_2012_37_MOESM12_ESM.tiff]
